# Dasatinib enhances antitumor activity of paclitaxel in ovarian cancer through Src signaling

**DOI:** 10.3892/mmr.2015.3784

**Published:** 2015-05-14

**Authors:** JUAN XIAO, MANMAN XU, TENG HOU, YONGWEN HUANG, CHENLU YANG, JUNDONG LI

**Affiliations:** 1 Department of Gynecology, Sun Yat-sen University Cancer Center, State Key Laboratory of Oncology in South China, Collaborative Innovation Center for Cancer Medicine, Guangzhou, Guangdong 510060; 2 Department of Obstetrics and Gynecology, The First Affiliated Hospital, Sun Yat-sen University, Guangzhou, Guangdong 510080; 3 Department of Gynecology, Affiliated Tumor Hospital, Xinjiang Medical University, Ürümqi, Xinjiang 830000, P.R. China

**Keywords:** Src, Src family tyrosine kinase, dasatinib, paclitaxel, ovarian cancer

## Abstract

Src family tyrosine kinase (SFK) activation is associated with ovarian cancer progression. Therefore, SFKs are targets for the development of potential treatments of ovarian cancer. Dasatinib is a tyrosine kinase inhibitor that targets SFK activity, and is used for the treatment of B cell and Abelson lymphomas. At the present time, the potential effect of dasatinib on ovarian cancer is not clear. The aim of the present study was to investigate the antitumor activity of dasatinib, alone and in combination with paclitaxel, in ovarian cancer *in vitro* and *in vivo*. In the present study, the expression of Src and phospho-Src-Y416 (p-Src) was measured in six ovarian cancer cell lines using western blotting and immunohistochemistry. In addition, cell viability and apoptosis were measured using an MTT assay and annexin V-fluorescein isothiocyanate staining. An ovarian cancer murine xenograft model was established, in order to evaluate the antitumor effect of dasatinib alone and in combination with paclitaxel in ovarian cancer. High levels of p-Src protein expression were observed in all cell lines, as compared with healthy cells, which indicated activation of the Src signaling pathway. p-Src expression increased in ovarian cancer cells following paclitaxel treatment. Dasatinib treatment demonstrated anti-ovarian cancer properties, by downregulating p-Src expression and by inducing cancer cell apoptosis. Combined treatment with dasatinib and paclitaxel markedly inhibited proliferation and promoted apoptosis of ovarian cancer cells, compared with control cells. Combined dasatinib and paclitaxel treatment exhibited antitumor activities *in vivo* and *in vitro* (combination indices, 0.25–0.93 and 0.31–0.75; and tumor growth inhibitory rates, 76.7% and 58.5%, in A2780 and HO8910 cell lines, respectively), compared with paclitaxel treatment alone. Dasatinib monotherapy demonstrated anti-ovarian cancer activities. The effects of dasatinib and paclitaxel treatments on ovarian cancer cells appeared to be mediated by the Src pathway.

## Introduction

Platinum and taxane-based chemotherapy following cyto-reductive surgery are the predominant approaches for the treatment of advanced ovarian cancer (1). Although the majority of patients respond well to primary chemotherapy, 75% will experience a relapse and acquire drug-resistance (1). Novel therapeutic strategies are, therefore, required in order to improve the prognosis for such patients (2). Dasatinib is one such therapy that targets the breakpoint cluster region-Abelson (BCR-ABL) fusion protein and the Src family tyrosine kinases (SFKs) (3). SFKs consist of nine non-receptor tyrosine kinases that share a similar structure (4). Cellular-Src kinase (c-Src) is expressed in a number of types of cells. It is involved in numerous signaling pathways, and in the formation and development of tumors (5). Overexpression and activation of c-Src has been observed in the development of certain solid tumors, such as breast, colon, prostate, ovarian, pancreatic and non-small cell lung cancer, and squamous cell carcinoma of the head and neck (6–10). Budde *et al* (11) detected Src expression in 60 human tumor cell lines and demonstrated that ovarian cancer cell lines exhibited a moderate level of Src expression, compared with healthy cell lines. A further study demonstrated Src overexpression and activation in advanced-stage ovarian tumor cells (12). Similarly, c-Src and phospho-Src-Y416 (p-Src; Tyr416) were shown to be overexpressed in human ovarian cancer cells (13). A number of different Src inhibitors have been analyzed in certain tumors. Dasatinib is a multi-targeted inhibitor of the receptor tyrosine kinases, Src and the BCR-ABL fusion protein (14). In June 2006, the Food and Drug Administration approved the use of dasatinib for the treatment of imatinib-resistant or imatinib-intolerant patients with chronic myeloid leukemia, and for the treatment of patients with Philadelphia-chromosome-positive acute lymphoblastic leukemia, who may be resistant or intolerant to first-line treatments (15). Dasatinib therapy has been investigated in other types of malignancies and the results observed for the treatment of solid tumors are encouraging. A number of studies have confirmed the effectiveness of dasatinib treatment for solid tumors (16–21), although few studies have focused on ovarian cancer. Therefore, the effects of dasatinib on ovarian cancer remain unclear. Konecny *et al* (22) examined the effects of dasatinib in 34 human ovarian cancer cell lines and demonstrated that 24/34 (71%) of representative ovarian cancer cell lines were highly sensitive to dasatinib. Furthermore, additive and synergistic interactions were observed, following treatment with dasatinib and carboplatin or paclitaxel. Similar results were presented by Teoh *et al* (23). However, the precise mechanisms underlying the antitumor effects of, and the interactions between, dasatinib and paclitaxel, such as cell survival, proliferation, autophagy, microtubule stability, motility and tumor angiogenesis remain unknown. The aim of the present study was to evaluate the antitumor properties of dasatinib alone and in combination with paclitaxel in ovarian cancer *in vitro* and *in vivo*. The mechanisms underlying the interactions between dasatinib and paclitaxel were also investigated.

## Materials and methods

### Drugs and reagents

In order to conduct an *in vitro* study, dasatinib (Selleck Chemicals, Houston, TX, USA) was dissolved in dimethylsulfoxide (DMSO; DaMao Chemical Reagent Factory, Tiangjin, China) at 10 mmol/l and stored at −20°C. Frequent freeze-thawing was avoided. In order to conduct an *in vivo* study, dasatinib was diluted in sterile distilled water at 1 mg/ml, and stored at 4°C for <7 days. Paclitaxel (Bristol-Myers Squibb, New York City, NY, USA) was diluted in 3 mg/ml sterile distilled water. The rabbit polyclonal anti-Src (cat. no. 2108S; 1:100) and rabbit polyclonal anti-phosphorylated Src (cat. no. 2101S; 1:60) antibodies were purchased from Cell Signaling Technology, Inc. (Danvers, MA, USA). The monoclonal mouse GAPDH antibody (cat. no KC-5G5; 1:1,000) was purchased from Kangchen (Shanghai, China). The goat-anti-rabbit secondary (cat. no. sc-2054; 1:1,000), and goat-anti-mouse secondary (cat. no. sc-2005; 1:1,000) antibodies were obtained from Santa Cruz Biotechnology, Inc. (Santa Cruz, CA, USA). A horseradish peroxidase (HRP) polymer-conjugated anti-rabbit secondary antibody (cat. no. PV-6001; 1:1,000) was purchased from ZSGB-BIO (Beijing, China), and Annexin V-fluorescein isothiocyanate (FITC) was obtained from Merck Millipore (Darmstadt, Germany). An Apoptosis Detection kit was purchased from EMD Millipore (Billerica, MA, USA). DMSO, MTT, and polyvinylidene difluoride (PVDF) membranes were purchased from Sigma-Aldrich (St. Louis, MO, USA).

### Cell lines and cell culture

The following six human ovarian cancer cell lines were used for analysis: A2780, HO8910, OVCAR3, CAOV3 and COC1 (Collection Conservation Center of Wuhan University, Wuhan, China), and SKOV3 (State Key Laboratory of Oncology in South China, Guangzhou, China). All cell lines were cultured in Dulbecco's modified Eagle's medium (DMEM; Thermo Fisher Scientific, Waltham, MA, USA), supplemented with 5% heat-inactivated fetal bovine serum (Guangzhou Ruite Bio-tec Co., Ltd., Guangzhou, China), penicillin (50 U/ml), and streptomycin (50 *µ*g/ml) (North China Pharmaceutical Co., Ltd., Shijiazhuang, China). Cells were grown in a humidified incubator with 5% CO_2_, at 37°C.

### Western blot analysis

Cells were cultured in six-well plates for 24 h and exposed to treatments when in the logarithmic growth phase. Cells were harvested and total protein was isolated. Total protein (20–40 *µ*g) was separated using electrophoresis, electrotransferred to PVDF membranes and then probed with the primary antibodies in Tris-buffered saline with Tween-20^®^ (TBST; Bioeasy Co., Ltd., Shenzhen, China)-5% milk (1:1,000) overnight, at 4°C. The membranes were incubated with a peroxidase-conjugated secondary antibody in TBST-5% milk (1:3,000) for 1–2 h, at room temperature. Protein detection was achieved using enhanced chemiluminescence reagent and XAR film (Kodak, Rochester, NY, USA) according to the manufacturer's instructions. Anti-GAPDH was used as a positive control (24).

### MTT assay

In order to measure cell viability and activity, cells in the logarithmic growth phase were seeded in 96-well plates (3,000 cells/well) and then treated with different drug concentrations. Concentrations were selected according to references (16,25,26) and preliminary experiments, which demonstrated that dasatinib (10 mg/kg) and paclitaxel (15 mg/kg) had marked anti-ovarian cancer effects. The following concentrations were used: Dasatinib (0.3125, 0.625, 1.25, 2.5, 5, 10 and 20 *µ*mol/l), paclitaxel (1.25, 2.5, 5, 10 and 20 *µ*mol/l) or a combination of dasatinib and paclitaxel (250:1 or 500:1). Negative control cells were treated with DMSO. Experiments were repeated three times. Following 72 h of drug treatment, 10 *µ*l of 5 mg⁄ml MTT was added to each well for an additional 4 h of incubation at 37°C, in 5% CO_2_. The medium was subsequently removed and 100 *µ*l DMSO was added to the cells. Plates were agitated gently for 10 min at 37°C and absorbance at 570 nm was measured using a microplate reader (Molecular Devices, West Berkshire, UK). Cytotoxicity was evaluated by calculating the 50% inhibitory concentration (IC_50_), and the additive or synergistic effects were assessed using combination indices (CI). CI<1 indicated synergy, CI=1 indicated additivity, and CI>1 indicated antagonism (24), calculated using Calcusyn 2.0 software (Biosoft, Cambridge, UK).

### Apoptosis assays

A2780 and HO8910 cell lines were cultured in six-well plates and treated with paclitaxel with or without dasatinib for 48–72 h in culture medium (37°C, 5% CO_2_). The cells were stained using an Annexin V-FITC Apoptosis Detection kit. Subsequently, cell cycle and apoptosis were measured at 488 nm, using flow cytometry (Beckman Coulter, Brea, CA, USA). Data were recorded as the percentage of cells in each phase of the cell cycle.

### Tumor xenografts

A total of 87 female BALB/c nude mice age, 5–6 weeks old, weight, 18–20 g, were obtained from Hunan SJA Lab Animal Co. Inc. (Hunan, China) and were maintained in specific pathogen-free conditions. Experiments were approved by the animal care committee of Sun Yat-sen University (Guangzhou, China) and were performed under sterile conditions. Mice were housed in Sun Yat-sen University Medical Animal Center and maintained in a 12 h light/dark cycle with access to food and water. A2780 or HO8910 cell lines (5×10^6^ cells) were subcutaneously injected into both side flanks, using a 23-gauge needle (each cell line was administered to two mice; Sun Yat-sun University Cancer Center). Once the tumors had reached a mean diameter of 8–10 mm, the mice were sacrificed and the tumor tissue was cut into 1–2-mm fragments in serum-free DMEM, at 4°C. The tumor fragments were subsequently transplanted subcutaneously into the flanks of recipient mice (42 mice were injected with the A2780 tumor fragments; 43 mice were injected with the HO8910 tumor fragments). The whole transplantation procedure was completed within 30–40 min. Tumor size and animal weight were monitored every 2–3 days. Once tumor volumes had reached 50–80 mm^3^ (mean diameter 3–5 mm, following 7 days of inoculation), mice were randomly divided into four groups of six, and treated intraperitoneally with one of the following: Dasatinib only (10 mg/kg, five times a week); paclitaxel only (15 mg/kg, once a week); combined dasatinib (10 mg/kg) and paclitaxel (15 mg/kg); or negative control (sterile distilled water). All treatments were administered for 4 weeks; dosage was derived from references (16,25,26) and preliminary experiments, which demonstrated that dasatinib (10 mg/kg) and paclitaxel (15 mg/kg) had marked anti-ovarian cancer effects. Tumor volumes were calculated using the formula: volume (mm^2^) = 0.5 x tumor length x tumor width^2^. Mice were sacrificed by cervical dislocation and tumor size, tumor weight, and mouse body weight were measured. Tumor tissues were excised and tissue samples were divided: One section was fixed in formalin (Guangzhou Chemical Reagent Factory, Guangzhou, China) for immunohistochemistry analysis and a second section was fixed in liquid nitrogen for western blotting. Tumor growth inhibitory rate (TIR) was calculated as follows: (average tumor weight in control group − average tumor weight in treatment group)/aver average tumor weight in control group × 100%.

### Immunohistochemistry

The xenograft tumor tissues were fixed in formalin, embedded in paraffin, sliced into 4-*µ*m sections, and rehydrated using a graded alcohol series. Endogenous peroxidase activity was blocked using 3% hydrogen peroxide (Guangzhou Chemical Reagent Factory) in methanol, for 10 min. For antigen retrieval, slides were heated in a pressure cooker in 10 mM citrate buffer (pH 6.0; Guangzhou Chemical Reagent Factory), for 10 min. The slides were incubated with anti-Src antibody (1:100) or anti-P-Src antibody (1:60) overnight, at 4°C. Slides were then incubated with an HRP polymer-conjugated anti-rabbit secondary antibody for 30 min, at 37°C and the color was developed using diaminobenzidine for 5 min. The nucleus was counter-stained using Meyer's hematoxylin (Guangzhou Chemical Reagent Factory). The negative control was obtained by replacing the primary antibody with normal rabbit immunoglobulin G. Human epithelial ovarian cancer slides were used as positive controls. Tumor tissue, fixed in liquid nitrogen, was pulverized in cell lysis buffer (1,000 *µ*l; Guangzhou Chemical Reagent Factory), on ice. Immediately, 1 mmol/l phenylmeth-anesulfonylfluoride (Guangzhou Chemical Reagent Factory) was added and the lysate was clarified twice by centrifugation at 12,000 × g and 4°C for 15 min. Western blotting was performed as described previously.

### Statistical analysis

Statistical analyses were performed using SPSS 17.0 (SPSS, Inc., Chicago, IL, USA). Student's t-test or Pearson's chi-squared tests were performed as appropriate. Analysis of variance (ANOVA) was used for repeated measurements (one-way ANOVA or Kruskal-Wallis test). P<0.05 was considered to indicate a statistically significant difference.

## Results

### Src and p-Src expression in ovarian cancer cell lines

**Figure 1:**
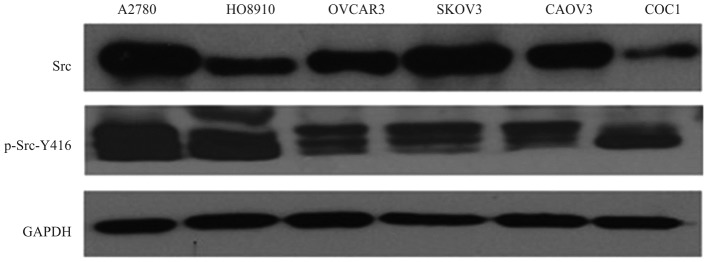
Expression of Src and p-Src (Tyr 416) in A2780, HO8910, OVCAR3, SKOV3, CAOV3, and COC1 type cell lines. p-Src, phospho-Src-Y416.

Src and p-Src expression levels were measured using western blotting. The six ovarian cancer cell lines exhibited high levels of Src and p-Src expression. A2780 and SKOV3 cells lines demonstrated the highest Src expression levels, whilst the A2780 and HO8910 cell lines demonstrated the highest p-Src expression levels (Fig. 1). Based on these results, the A2780 and HO8910 cell lines were selected for further analysis.

### Paclitaxel activates the Src pathway in ovarian cancer cells

**Figure 2:**
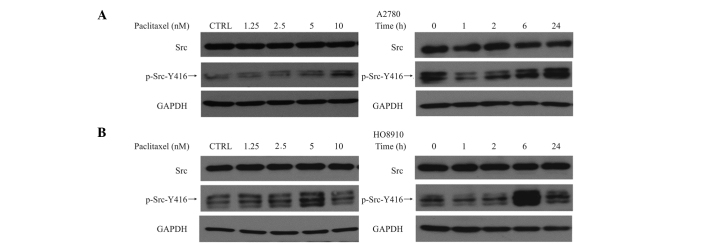
Src expression and phosphorylation in A2780 and HO8910 cell lines. (A) A2780 cells treated with different concentrations of paclitaxel for 6 h (left) and 10 nM paclitaxel for different times (right); (B) HO8910 cells treated with different concentrations paclitaxel for 6 h (left) and 10 nM paclitaxel for different times (right). CTRL, control; p-Src, phospho-Src-Y416.

p-Src expression was upregulated in a dose-dependent manner. However, total Src protein expression was unaffected, in A2780 and HO8910 cell lines, following treatment with paclitaxel. For the A2780 cells, p-Src expression was greatest following 24 h of treatment with 10 nmol/l paclitaxel. For HO8910 cells, p-Src expression was greatest following 6 h of treatment with 5 nmol/l (Fig. 2).

### Dasatinib inhibits viability and enhances paclitaxel cytotoxicity in ovarian cancer cells

**Figure 3:**
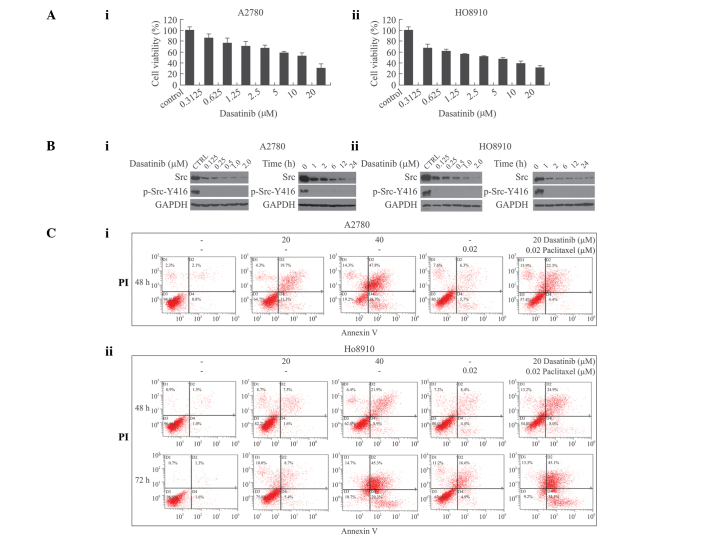
Dasatinib enhanced the paclitaxel-associated cytotoxicity in ovarian cancer cells. (A) Cell death following treatment with dasatinib for 72 h, in A2780 and HO8910 cell lines. Cell death was determined using an MTT assay. The IC_50_ of dasatinib was 7.30±0.64 *µ*M in A2780 cells and 2.51±0.64 *µ*M in HO8910 cells. (B) Src expression and phosphorylation in A2780 and HO8910 cell lines. (Ba) Left, A2780 cells treated with dasatinib for 2 h; right, A2780 cells treated with 0.1 *µ*M dasatinib over time. (Bb) Left, HO8910 cells treated with dasatinib for 2 h; right, HO8910 cells treated with 0.1 *µ*M dasatinib over time. Control cells were treated with 0.02% dimethyl sulfoxide. (C) Dasatinib enhanced cell apoptosis and enhanced paclitaxel-induced cytotoxicity in ovarian cancer cells. (Ca) A2780 cells treated with 20 and 40 *µ*M dasatinib for 48 h exhibited apoptosis rates of 30.9±1.4 and 66.5±0.9% of the control, respectively. (Cb) HO8910 cells treated with 20 and 40 *µ*M dasatinib for 48 h exhibited apoptosis rates of 8.65±0.6 and 30.8±1.2% of the control, respectively. At 72 h the rates were 12.6±2.2 and 76.0±2.0% of the controls, respectively. (P<0.05). p-Src, phospho-Src-Y416.

The results of the present study demonstrated that paclitaxel treatment activated Src expression in ovarian cancer cells. Subsequently, the effect of dasatinib on the cytotoxicity of paclitaxel was investigated in these cell lines. A2780 and HO8910 cell lines treated with increasing dasatinib concentrations (0.3125-20 *µ*M) demonstrated a dose-dependent reduction in cell viability (Fig. 3A). IC_50_ values were 7.30±0.64 *µ*M in A2780 cells and 2.51±0.64 *µ*M in HO8910 cells. Cell lines were sensitive to paclitaxel treatment (IC_50_~20 nmol/l). Dasatinib combined with paclitaxel demonstrated synergistic antitumor activity with confidence interval (CI) values of 0.25–0.93 and 0.31–0.75 in A2780 and HO8910 cell lines, respectively (Table I). In addition, at concentrations below the IC_50_, dasatinib suppressed Src and p-Src protein expression (Fig. 3B). An annexin V-FITC/propidium iodide apoptosis assay was conducted in order to determine whether the cytotoxicity of dasatinib, with or without paclitaxel treatment, is associated with changes in cell apoptosis. Apoptosis rates in A2780 cells treated with 20 *µ*M and 40 *µ*M dasatinib for 48 h, were 30.9±1.4 and 66.5±0.9%, respectively. In HO8910 cells, these rates were 8.65±0.6 and 30.8±1.2% following 48 h of treatment, and 12.6±2.2 and 76.0±2.0% following 72 h of treatment, respectively (P<0.05). A greater percentage of apoptotic cells was observed following treatment with dasatinib combined with paclitaxel, compared with cell treated with dasatinib only. The apoptotic rates of A2780 cells following treatment with 20 *µ*M dasatinib, 0.02 *µ*M paclitaxel, and combined dasatinib and paclitaxel, for 48 h, were 34.7±3.4, 20.9±3.7 and 44.2±2.4%, respectively. Apoptotic rates were 17.3±2.1, 22.0±2.7 and 43.6±4.0% in HO8910 cells (all P<0.001 compared with control cells; Fig. 3C).

### Antitumor activity of combined dasatinib and paclitaxel in a human ovarian cancer xenograft model

**Figure 4:**
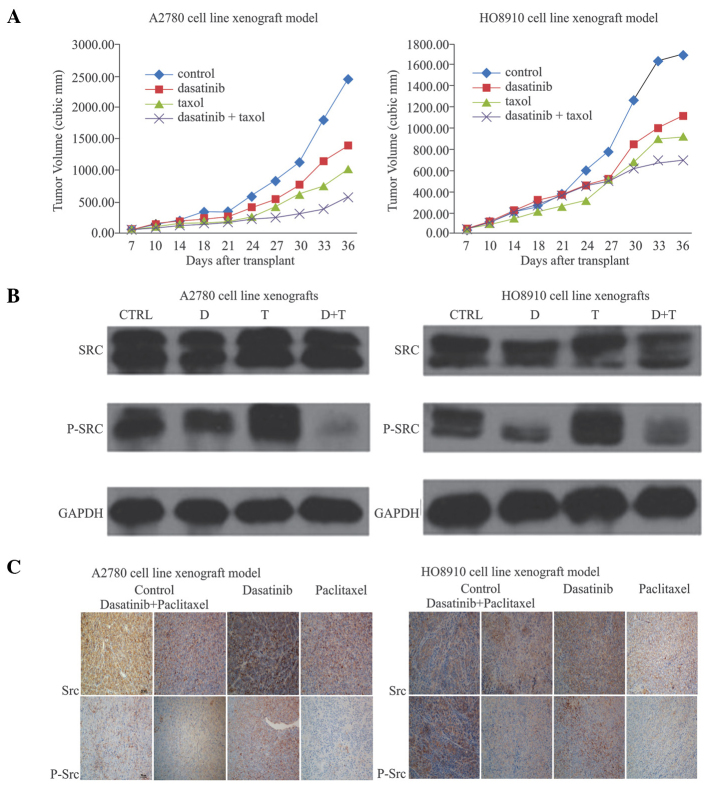
Antitumor activity of combined dasatinib and paclitaxel in a human ovarian cancer xenograft model. (A) Ovarian cancer xenograft growth curve. Mice bearing ovarian cancer xenografts were treated as follows: Dasatinib alone (10 mg/kg five times a week); paclitaxel alone (15 mg/kg once a week); combination of dasatinib (10 mg/kg per week); and paclitaxel (15 mg/kg per week); vehicle control (the same quantity of sterile distilled water). P=0.003, dasatinib vs. control in A2780 cells; P=0.004, combined treatment vs. dasatinib alone in A2780 cells; P=0.087, combined treatment vs. paclitaxel in A2780 cells; P=0.024, dasatinib vs. control in HO8910 cells; P=0.075, combined treatment vs. dasatinib alone in HO8910 cells; P=0.281, combined treatment vs. paclitaxel in HO8910 cells. Group-by-time interaction for tumor growth was statistically significant (A2780, F=8.054 and P<0.001; HO8910, F=7.681 and P<0.001). (B) Western blot analysis of Src and p-Src protein *in vivo*. (C) Immunohistochemistry of Src and p-Src protein expression in ovarian cancer xenografts. p-Src, phospho-Src-Y416; CTR, control; D, dasatinib; T, paclitaxel.

A2780 and HO8910 xenografts were established in nude mice in order to assess the potential antitumor effect of treatment with dasatinib alone or with dasatinib in combination with paclitaxel. In A2780 and HO8910 xenografts, dasatinib treatment led to tumor growth inhibition by 43.2% (A2780) and 34.0% (HO8910; Table II; Fig. 4A). Combined treatment led to a greater growth inhibitory effect: TIRA2780 = 76.7% (P<0.001) and TIRHO8910 = 58.5% (P<0.001) in comparison with single drug treatments. The comparison of tumor-growth curves, using group and time as variables, with a two-sided ANOVA, demonstrated that the group-by-time interaction for tumor growth (F) was statistically significant (F_A2780_ = 8.054, P_A2780_<0.001; FHO8910 = 7.681, PHO8910 < 0.001). As shown in Table II, mouse weight was not significantly different between groups (one-way ANOVA: FA2780 = 5.619, PA2780 = 0.162; FHO8910 = 0.6, PHO8910 = 0.794). Overall, treatments appeared to be relatively well tolerated by the mice, with no mortalities or weight loss, and no signs of acute or delayed toxicity. Western blotting (Fig. 4B) and immunohistochemistry (Fig. 4C) suggested that paclitaxel activated and upregulated the Src pathway (p-Src expression) in cancer cells, compared with that in the control cells. Dasatinib combined with paclitaxel treatment downregulated p-Src expression in cancer cells (23.3 vs. 31.0% in A2780 cells and 21.3 vs. 48.7% in HO8910 cells), as compared with dasatinib treatment alone (Figs. 4B and C). This is consistent with the results of the *in vitro* study.

## Discussion

Ovarian carcinoma is the leading cause of reproductive-associated cancer mortality among females worldwide (1). Despite high initial remission rates using paclitaxel and platinum-based chemotherapy, <75% of patients with advanced-stage ovarian carcinoma will relapse (1). Targeted agents have been the primary focus of treatment efforts for patients with recurrent ovarian cancer. A number of studies have aimed to investigate novel antitumor drugs that interfere with the critical signaling pathways associated with the occurrence and progression of ovarian cancer. Dasatinib is a competitive inhibitor of a number of tyrosine kinases, including SFKs (27). A number of studies have suggested that dasatinib alone or in combination with other drugs, such as paclitaxel, exhibits preclinical antitumor activity in human ovarian cancer (22,23,25). However, the synergistic mechanisms associated with dasatinib treatment combined with paclitaxel, remain unknown. The results of the present study suggested that dasatinib treatment enhanced the paclitaxel-associated antitumor activities in ovarian cancer cells. This may be explained by the inhibitory effects of dasatinib on the Src signal pathway.

Mayer and Krop (28) demonstrated that Src regulates a number of signaling pathways that affect proliferation, survival, migration, invasion and angiogenesis in tumor cells, and that Src is activated and overexpressed in numerous types of solid tumors (6–10,16,26,29). The current study demonstrated overexpression of Src and p-Src in human ovarian cancer tissues (13). In the present study, the Src signaling pathway was activated, and p-Src was overexpressed in six human ovarian cancer cell lines. Given the effect of Src signaling on ovarian cancer, Konecny *et al* (22) studied 34 ovarian cancer cell lines and demonstrated that dasatinib inhibits cell proliferation *in vitro* with 0.001–11.3 *µ*M IC_50_ values. In the current study, dasatinib was found to inhibit ovarian cancer cell proliferation. Low concentration dasatinib (IC_50_~8 *µ*M) reduced cell proliferation, and Src and p-Src protein expression levels were downregulated in response to dasatinib treatment at varying concentrations and incubation times. Therefore, dasatinib cytotoxicity in ovarian cancer may be explained by the inhibition of Src pathway activation. Teoh *et al* (23) suggested that dasatinib caused a reduction in p-Src expression in ovarian cell lines. The results of the present study confirm these findings. Furthermore, the results of the current study suggested that paclitaxel activated Src signaling in ovarian cancer cells. In the current study, p-Src expression was upregulated in A2780 and HO8910 cell lines following treatment with different concentrations of paclitaxel. However, Src protein expression was not upregulated. These results were confirmed in the *in vivo* studies. Based on the results of the present study, it is hypothesized that primary and acquired resistance to paclitaxel in ovarian cancer may be due to the activation of the Src signaling pathway, and the associated cancer cell proliferation, angiogenesis, invasion and dissemination.

A significant synergetic antitumor effect was observed following treatment with a combination of dasatinib and paclitaxel, which was associated with inhibition of Src pathway activity. In accordance with the results of the *in vitro* study, dasatinib suppressed the growth of ovarian cancer xenografts in nude mice compared with the controls (TIR 43.2% in A2780 cells, and 34.0% in HO8910 cells, both P<0.01). Furthermore, when combined with paclitaxel, a synergistic antitumor effect was observed *in vitro* (CI<1; TIR values were 76.7 and 58.5% in A2780 cells and HO8910 cells, respectively, P<0.05). The synergy between dasatinib and paclitaxel was associated with the inhibition of p-Src protein expression. These results suggested that paclitaxel exhibits antitumor activity in ovarian cancer, but may also activate the Src signal pathway, which promotes cell survival and drug resistance.

The mechanisms underlying dasatinib-enhanced antitumor activity and the association with paclitaxel, remain unclear. SFK inhibitors enhance paclitaxel sensitivity by inducing cell apoptosis, autophagy, microtubule stability and neovasculature (30). George *et al* (31) demonstrated that Src inhibition reduces the critical intracellular concentration at which pacli-taxel induces tubulin stabilization and apoptosis. Furthermore, Chen *et al* (32) demonstrated that Src inhibition alone activates caspase-3 and promotes apoptosis. The results of the present study suggested that dasatinib promoted apoptosis in a mouse xenograft model. The results suggested that dasatinib exhibited a dose- and time-dependent effect on the level of apoptosis in ovarian cancer cell lines (both P<0.05). Therefore, dasatinib is capable of enhancing apoptosis following paclitaxel treatment.

The use of additive targets in cancer therapy, such as the use of targeted agents in combination with cytotoxic drugs, is becoming increasingly common. Ovarian cancer is highly responsive to first-line chemotherapy, following optimal debulking surgery. However, relapses are frequently observed in patients, due to the development of resistance to conventional chemotherapy drugs. Therefore, combination therapy may be useful for the treatment of ovarian cancer (33). Novel therapeutic agents in combination with conventional therapies may prevent the emergence of resistance, thereby prolonging remission and improving long-term survival. In the present study, a synergistic effect was observed following treatment with a combination of dasatinib and paclitaxel, *in vitro* and *in vivo*. A number of studies have demonstrated that SFK inhibition sensitizes cancer cells to paclitaxel-based treatments by modulating cell survival and proliferation, autophagy, microtubule stability, motility, and tumor angiogenesis (23,32,34–36). Numerous mechanisms have been proposed in order to explain these observations.

The present study demonstrated that p-Src expression levels were markedly increased following paclitaxel treatment. Therefore, paclitaxel activates Src signaling. However, dasatinib treatment appeared to suppress p-Src expression, *in vitro* and *in vivo*. Similarly, a separate study demonstrated that oxaliplatin activates Src signaling via a reactive oxygen species-dependent mechanism, and a trend was observed between the degree of Src activation following oxaliplatin treatment and the degree of synergy between dasatinib and oxaliplatin (37). Furthermore, acquired and persistent resistance to paclitaxel is associated with upregulation of Multi-Drug Resistance (MDR)-1, increased DNA damage tolerance and drug metabolism, altered micro-tubule isotype expression and mutations of β-tubulin (31). The inhibition of certain SFKs eliminated the resistance of ovarian cancer cells via an MDR-independent mechanism. However, the pathways underlying this process remain unclear (31). A study has shown that dasatinib may enhance paclitaxel sensitivity by suppressing B cell lymphoma-2 and cyclin dependent kinase 1 expression in ovarian cancer cells via a p27(Kip1)-dependent process (34). The mechanisms involved in the paclitaxel-activated Src pathway are unclear. However, it is clear that this activation is a primary factor in the synergistic effects of combined dasatinib and paclitaxel treatment in ovarian cancer. The present study suggested that dasatinib increased the cytotoxicity of paclitaxel in ovarian cancer, possibly a result of dasatinib-driven inhibition of paclitaxel-induced Src activity. Therefore, in response to dasatinib treatment, apoptosis was enhanced and paclitaxel resistance was delayed in ovarian cancer cells.

The preclinical data in the present study suggests that dasatinib is a potential therapeutic agent for ovarian cancer. When combined with paclitaxel, dasatinib is able to further enhance anti-ovarian cancer activity, and may delay or prevent paclitaxel resistance.

## Figures and Tables

**Table I:** Effects of dasatinib combined with paclitaxel.

A, A2780 ovarian cancer cell line			

Dasatinib (*µ*M)	Paclitaxel (*µ*M)	Fa	CI
0.3125	0.0013	0.4089	0.330
0.6250	0.0025	0.4247	0.575
1.2500	0.0050	0.4650	0.817
2.5000	0.0100	0.5277	0.973
5.0000	0.0200	0.6903	0.483
10.000	0.0400	0.8178	0.245

B, HO8910 ovarian cancer cell line			

Dasatinib (*µ*M)	Paclitaxel (*µ*M)	Fa	CI
0.3125	0.0006	0.4713	0.402
0.625	0.0013	0.5130	0.526
1.25	0.0025	0.5500	0.746
2.5	0.0050	0.7395	0.308
5.0	0.0100	0.8294	0.257
10.0	0.0200	0.8229	0.553

CI, confidence interval; Fa, inhibition rate.

**Table II:** Body weight, tumor weight and tumor volume in ovarian cancer xenografts.

A, A2780 xenografts						

Treatment	n	Weight (g)		Tumor volume	Tumor weight	TIR
		Day 0	Day 31			
Control	5/8	20.88±0.18	22.47±0.89	2465.0±459.1	2.12±0.34	
Dasatinib	6/12	20.93±0.72	22.37±0.89	1400.2±231.3	1.67±0.30	43.197
Paclitaxel	6/11	21.17±0.45	23.08±0.72	1016.9±176.3	1.34±0.20	58.764
Combined	6/11	21.25±0.41	21.7±1.1	574.3±126.1	0.93±0.19	76.702

B, HO8910 xenografts						

Treatment	n	Weight (g)		Tumor volume	Tumor weight	TIR
		Day 0	Day 31			
Control	6/11	18.30±0.60	22.32±0.40	1695.2±162.5	2.65±0.28	
Dasatinib	6/10	19.10±0.66	20.88±0.58	1119±168.6	1.55±0.30	33.99
Paclitaxel	6/12	19.45±0.66	20.57±0.88	923.2±139.9	1.54±0.22	45.54
Combined	6/10	17.28±0.51	20.35±0.57	698.3±145.7	1.65±0.42	58.51

Tumor volume (mm^3^); tumor weight (g); TIR (%). Data are presented as the mean ± standard deviation. TIR, tumor growth inhibitory rates.
